# A High-Methionine Diet for One-Week Induces a High Accumulation of Methionine in the Cerebrospinal Fluid and Confers Bipolar Disorder-like Behavior in Mice

**DOI:** 10.3390/ijms23020928

**Published:** 2022-01-15

**Authors:** Isao Ishii, Shotaro Kamata, Saki Ito, Aya Shimonaga, Maika Koizumi, Maiko Tsushima, Asumi Miura, Tomoko Nagata, Yuka Tosaka, Haruka Ohtani, Waka Kamichatani, Noriyuki Akahoshi

**Affiliations:** 1 Department of Health Chemistry, Showa Pharmaceutical University, Tokyo 194-8543, Japan; kamata@ac.shoyaku.ac.jp (S.K.); a16016@ug.shoyaku.ac.jp (S.I.); b16052@ug.shoyaku.ac.jp (A.S.); b17044@ug.shoyaku.ac.jp (M.K.); b17076@ug.shoyaku.ac.jp (M.T.); kajiwara@ac.shoyaku.ac.jp (W.K.); akahoshi@ac.shoyaku.ac.jp (N.A.); 2 Department of Biochemistry, Keio University Faculty of Pharmacy, Tokyo 105-8512, Japan; b17112@ug.shoyaku.ac.jp (A.M.); a17024@ug.shoyaku.ac.jp (T.N.); a17085@ug.shoyaku.ac.jp (Y.T.); d2103@g.shoyaku.ac.jp (H.O.)

**Keywords:** amino acid, methionine, homocysteine, cerebrospinal fluid, behavior, bipolar disorder, open-field test, Y-maze test

## Abstract

Methionine (Met) is considered the most toxic amino acid in mammals. Here, we investigated biochemical and behavioral impacts of ad libitum one-week feeding of high-Met diets on mice. Adult male mice were fed the standard rodent diet that contained 0.44% Met (1×) or a diet containing 16 graded Met doses (1.2×–13×). High-Met diets for one-week induced a dose-dependent decrease in body weight and an increase in serum Met levels with a 2.55 mM peak (versus basal 53 µM) on the 12×Met diet. Total homocysteine (Hcy) levels were also upregulated while concentrations of other amino acids were almost maintained in serum. Similarly, levels of Met and Hcy (but not the other amino acids) were highly elevated in the cerebrospinal fluids of mice on the 10×Met diet; the Met levels were much higher than Hcy and the others. In a series of behavioral tests, mice on the 10×Met diet displayed increased anxiety and decreased traveled distances in an open-field test, increased activity to escape from water soaking and tail hanging, and normal learning/memory activity in a Y-maze test, which were reflections of negative/positive symptoms and normal cognitive function, respectively. These results indicate that high-Met ad libitum feeding even for a week can induce bipolar disorder-like disease models in mice.

## 1. Introduction

Methionine (Met) is one of the nine essential amino acids in mammals but is also the most toxic proteinogenic amino acid, whether expressed in terms of percentage of the diet or an increase over the nutritional requirement [1,2]. Excessive Met in the diet causes various deleterious alterations in experimental animals, including the suppression of food intake and near-cessation of growth, schizophrenic symptoms, enlarged spleen/kidney/liver, and hemosiderosis/hemolytic anemia [2,3,4,5,6,7]. Furthermore, Met administration is known to exacerbate psychopathological symptoms among schizophrenic patients [8] and to induce hippocampal alteration associated with memory deficits, schizophrenia-like social deficits, and pre-pulse inhibition impairment in mice [5,6,9]; the molecular mechanisms for these Met effects remain unknown.

Met might be indirectly responsible for deleterious effects in human cases because no adverse symptoms are observed in hypermethioninemia patients due to innate Met adenosyltransferase (MAT) I/III deficiency (OMIM 250850) [10]. Met is metabolized by methionine adenosyltransferase (MAT) to *S*-adenosylmethionine (SAM), the most predominant methyl donor for hundreds of methyltransferases. The enhancement of methylation reactions could be associated with unique toxicity, especially via negative transcription regulation, because glycine supplementation often alleviates the Met toxicity (probably through its self-sacrificing methylation to sarcosine [*N*-methylglycine] by glycine *N*-methyltransferase) [4,11]. SAM is thereafter metabolized to *S*-adenosylhomocysteine (SAH), and then SAH is hydrolyzed to homocysteine (Hcy), a highly toxic/reactive metabolite that plays a major detrimental role in cardiovascular disease [12,13,14,15]. Therefore, total Hcy in the blood is normally maintained at low levels (under 10 µM) by prompt remethylation to Met by Met synthase and betaine-Hcy methyltransferase, or converted to cysteine (Cys) via the transsulfuration pathway [7,10].

Moreover, Met could have competitive effects on amino acid transport [1]. Met is incorporated into cells via the amino acid transport systems including System A, System b^0,+^, System B^0,+^, System L, and System y^+^L [16]. Therefore, elevated levels of Met, which have generally higher affinities to those transporters, might interfere with the cellular incorporation of other amino acids that share the transporters. Indeed, we previously observed high urinary excretion of Met as well as several other neutral amino acids including five essential ones (valine, leucine, isoleucine, phenylalanine, and threonine) in hypermethioninemic mice lacking cystathionine β-synthase (CBS), one of the transsulfuration enzymes [17]. In addition, excessive Met could be metabolized by transamination, leading to the production of some highly toxic metabolites, ammonia, methylthiopropinate, and methanethiol, or by *S*-oxidation to a highly oxidative methionine sulfoxide [9], which was the most apparent in mice lacking cystathionine γ-lyase (CTH), another transsulfuration enzyme [18].

In this study, we investigated the biochemical and behavioral impacts of one-week diets with graded doses of Met on adult C57BL/6J mice. Serum and cerebrospinal fluid (CSF) levels of Met, total Hcy, and other amino acids were measured and the impacts of the diets on general mice behaviors were investigated. In addition, the influences of innate transsulfuration impairments (heterozygous *Cbs* or *Cth* deletion) in mice on serum Met/Hcy levels were evaluated. Our results demonstrate that one-week high-Met diets on mice drastically alter their circulatory/CSF amino acid profiles as well as their behavior.

## 2. Results

### 2.1. Altered Serum Amino Acid Profile on the High-Met Diets

**Figure 1:**
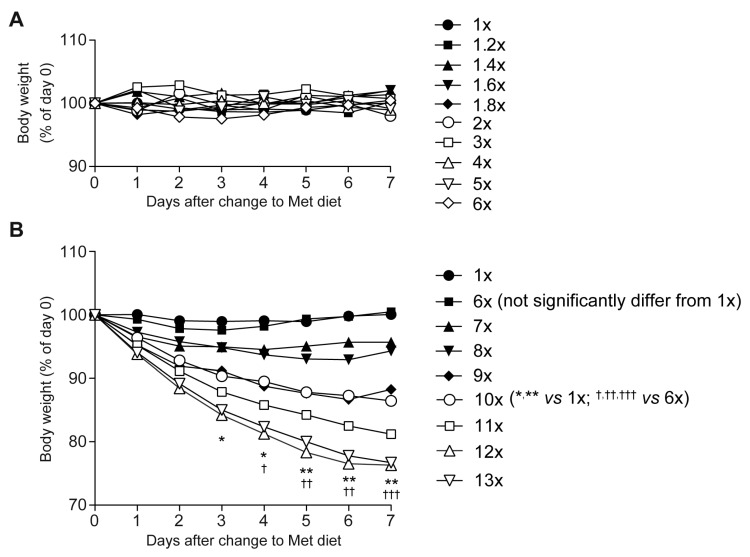
Impact of one-week high-methionine (Met) diet on body weight. Adult C57BL/6J male mice were ad libitum fed a standard CE-2 rodent diet (0.440% Met) until 8–9 weeks of age and then fed CE-2 (1×Met) or CE-2 containing graded concentrations of Met [1.2×Met [0.528%]–6×Met [2.64%] (**A**); 6×Met [2.64%]–13×Met [5.72%] (**B**)] for a week. Body weights were measured daily at 11 a.m. Data are the mean of four mice. Bodyweight decreases were evident with the 7×Met–13×Met diet. Statistical analysis was done by one-way ANOVA with a Tukey’s multiple comparison test at each time point. For clarity, only analyses between 1×Met, 6×Met, and 10×Met samples are shown; the differences were significant in * *p* < 0.05 and ** *p* < 0.01 versus 1×Met; ^†^ *p* < 0.05, ^††^ *p* < 0.01, and ^†††^ *p* < 0.001 versus 6×Met.

C57BL/6J male mice (8–10 weeks old) were ad libitum fed a standard/control (1×Met [0.44%]) or high-Met diets (1.2× [0.528%]–13× [5.72%]) for a week with free access to water. Body weights were not largely altered on the 1.2×Met–6×Met diets (Figure 1A), but they did gradually decrease day by day with the 7×Met–13×Met diets in a dose-dependent manner, reaching a maximal 24% reduction in a week with the 12×Met or 13×Met diet (Figure 1B). However, even at such severe health status, serum biochemical parameters, including aspartate aminotransferase (AST), alanine aminotransferase (ALT), lactate dehydrogenase (LDT), glucose (GLU), blood urea nitrogen (BUN), triglyceride (TG), and malondialdehyde (MDA), were generally normal, except some parameters on the 10×Met–13×Met diets (Supplementary Table S1).

**Figure 2:**
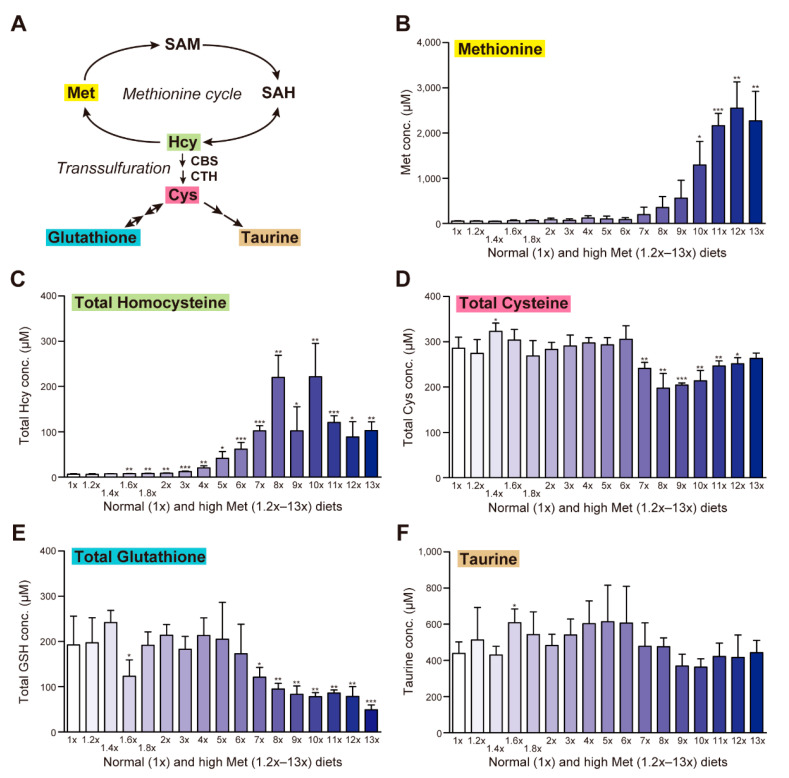
Impact of one-week high-methionine (Met) diet on serum levels of Met and cysteine (Cys) metabolites. (**A**) Schematic representation of the Met metabolic pathway (Met cycle, transsulfuration, and Cys metabolism) in mammals. (**B**–**F**) Adult C57BL/6J male mice were ad libitum fed a standard CE-2 rodent diet (0.440% Met) until 8–9 weeks of age and then fed CE-2 (1×Met) or CE-2 containing graded concentrations of Met (1.2×Met [0.528%]–13×Met [5.72%]) for a week. Serum levels of Met (**B**), total homocysteine (**C**), total Cys (**D**), total glutathione (**E**), and taurine (**F**) were measured using HPLC. Data are the mean ± SD (*n* = 8 for 1×Met and 6×Met and *n* = 4 for the others). The differences versus 1×Met samples were significant at * *p* < 0.05, ** *p* < 0.01, and *** *p* < 0.001 in the Student’s *t*-test.

Intestinally absorbed Met is converted to Hcy in the liver via the Met cycle (also known as the re-methylation cycle) and then to Cys via a transsulfuration that is composed of sequential enzymatic reactions catalyzed by CBS and CTH (Figure 2A). Thereafter, Cys is converted to the two major antioxidants, taurine and glutathione (GSH). Conversion to taurine is a one-way reaction, but conversion to GSH is reversible; therefore, GSH is considered safe cellular storage for the highly reactive Cys (Figure 2A). The high-Met diets induced dose-dependent Met accumulation in serum, reaching a peak on the 12×Met diet (2.55 mM; 48.3-fold higher than the basal level of 52.9 µM) (Figure 2B). In contrast, the same diets caused significant elevation of total Hcy levels in serum as low as 1.6×Met, reaching a peak around 220 µM with the 8×Met or 10×Met diet (Figure 2C). The levels of total Cys, total GSH, and taurine were not altered with the 1.2×Met–6×Met diets; however, those levels decreased (though not significantly for taurine) with the 7×Met–13×Met diets (Figure 2D–F, respectively).

**Figure 3:**
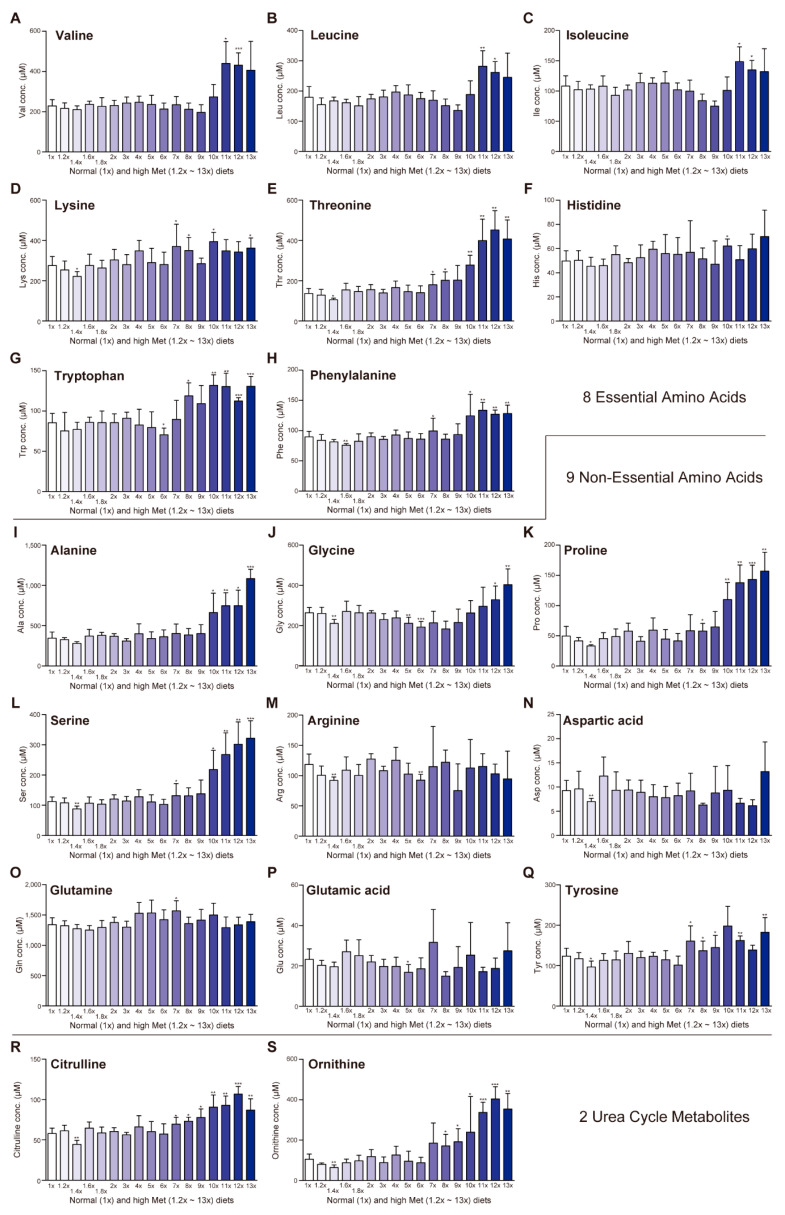
Impact of one-week high-methionine (Met) diet on serum amino acid levels. Adult C57BL/6J male mice were ad libitum fed a standard CE-2 rodent diet (0.440% Met) until 8–9 weeks of age and then fed CE-2 (1×Met) or CE-2 containing graded concentrations of Met (1.2×Met [0.528%]–13×Met [5.72%]) for a week. Serum levels of eight essential amino acids [valine (**A**), leucine (**B**), isoleucine (**C**), lysine (**D**), threonine (**E**), histidine (**F**), tryptophan (**G**), and phenylalanine (**H**)], nine non-essential amino acids [alanine (**I**), glycine (**J**), proline (**K**), serine (**L**), arginine (**M**), aspartic acid (**N**), glutamine (**O**), glutamic acid (**P**), and tyrosine (**Q**)], and two urea cycle metabolites [citrulline (**R**) and ornithine (**S**)] were measured using HPLC. Data are the mean ± SD (*n* = 8 for 1×Met and 6×Met and *n* = 4 for others). The differences versus 1×Met samples were significant at * *p* < 0.05, ** *p* < 0.01, and *** *p* < 0.001 in the Student’s *t*-test.

Serum levels of the other eight essential amino acids were generally maintained with the 1.2×Met–8×Met diets; however, those levels were slightly upregulated on the 9×Met–13×Met diets (Figure 3A–H). Similarly, serum levels of some non-essential amino acids (alanine, glycine, proline, and serine) were maintained with the 1.2×Met–9×Met diets but were somewhat upregulated with the 10×Met–13×Met diets (Figure 3I–L). In contrast, serum levels of arginine, aspartic acid, glutamine, glutamic acid, and tyrosine were not significantly altered by any of the tested high-Met diet (Figure 3M–Q). In addition, serum levels of two urea cycle metabolites, citrulline and ornithine, were upregulated on the 7×Met (or 8×Met)–13×Met diets, perhaps reflecting the upregulation of the Met deamination pathway [18].

### 2.2. Hyperaccumulation of Met in the CSF on the 10×Met Diet

**Figure 4:**
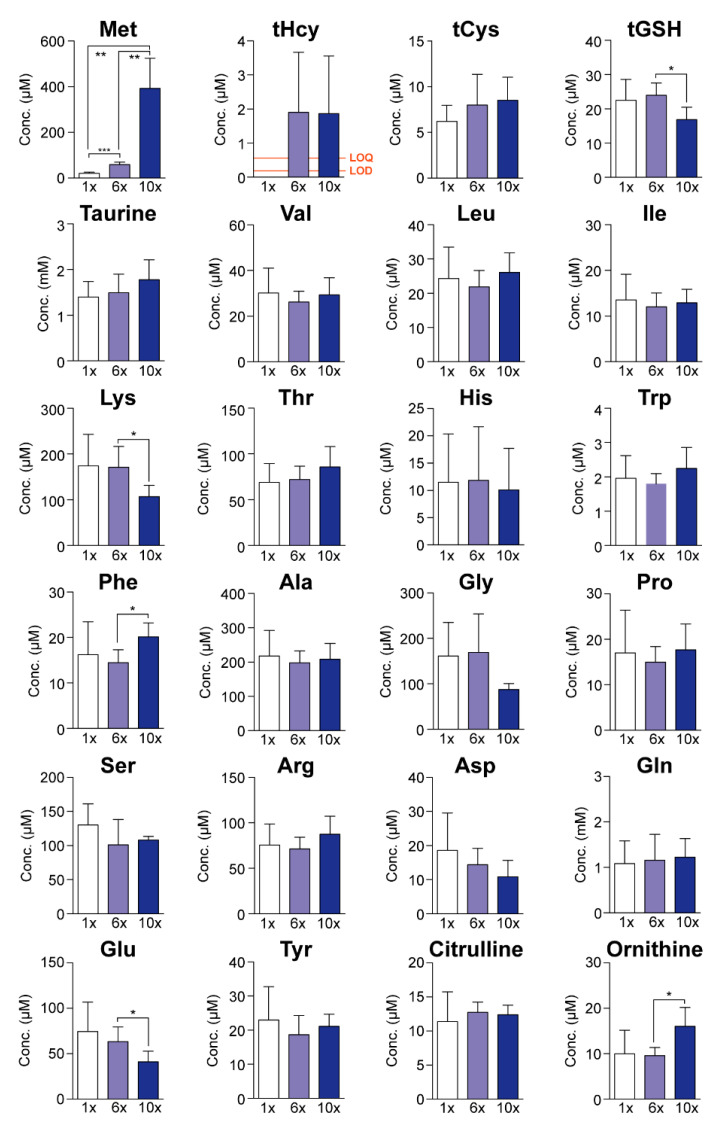
Impact of one-week high-methionine (Met) diet on cerebrospinal fluid (CSF) amino acid levels. Adult C57BL/6J male mice were ad libitum fed a standard CE-2 rodent diet (0.440% Met) until 8–9 weeks of age and then fed a standard (1×Met), 6×Met [2.64%], or 10×Met [4.40%] diet for a week. CSF levels of 17 amino acids, total homocysteine and glutathione, taurine, citrulline, and ornithine were measured using HPLC. Data are the mean ± SD (*n* = 5 each). The differences versus 1×Met samples were significant at * *p* < 0.05, ** *p* < 0.01, and *** *p* < 0.001 in the Student’s *t*-test. LOD, limit of detection; LOQ, limit of quantitation.

We had previously reported that Met accumulated highly in the CSF of 2-week-old CBS-deficient mice (488 µM compared to 24 µM in wild-type mice), an animal model of homocystinuria/homocysteinemia [19], and that adult CBS-deficient mice displayed cerebellar malformation and impaired learning ability [20]. Therefore, we next investigated the influence of the high-Met diet on the CSF amino acid profile. Because of their very low yields by ventricular puncture, the CSF samples from 3–4 mice were pooled, and their amino acid concentrations were measured. The CSF levels of Met were 53.9 ± 7.3 µM on the 6×Met diet and 364 ± 163 µM on the 10×Met diet, compared to 19.3 ± 5.3 µM on the control diet (Figure 4). In addition, CSF levels of total Hcy were as high as 2.66 ± 0.66 µM on the 6×Met diet and 2.59 ± 0.51 µM on the 10×Met diet, compared to levels in the control that were under the limit of detection (LOD) [0.16 µM] and the limit of quantitation (LOQ) [0.54 µM]. However, all the CSF Hcy levels were much lower than the CSF Met levels (Figure 4). There was no alteration in other CSF amino acid concentrations, except for slight (but significant) increases in phenylalanine/ornithine and decreases in total GSH/lysine/glutamic acid on the 10×Met diet (Figure 4).

### 2.3. Behavioral Alterations

**Figure 5:**
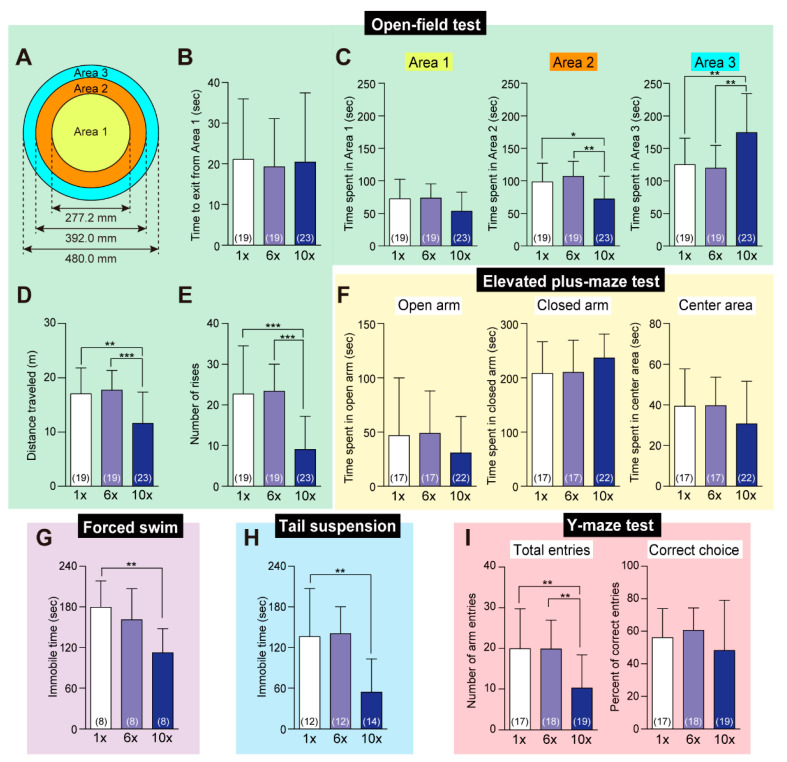
Behavioral assessments. Behavioral analyses were done on mice fed the control (1×Met), 6×Met, or 10×Met diet for a week. (**A**–**E**) Open-field test. Each mouse could roam the circular flat field with a side wall (**A**) consisting of Areas 1–3 (equal dimensions) for 5 min. Time to exit Area 1 (**B**), retention times in each area (**C**), distance traveled (**D**), and the number of rises (**E**) were measured. (**F**) Elevated plus-maze test. Retention times in the open arm, closed arm, and the center area was measured in the 5-min test. (**G**) Forced swim test. Each mouse was forced to swim for 7 min and the immobility time was recorded. (**H**) Tail suspension test. Each mouse was suspended by its tail for 6 min and the immobility time was recorded. (**I**) Y-maze test. Each mouse could roam the Y-maze apparatus for 5 min, and total arm entry times and percentages of correct arm choices were counted. Data are the mean ± SD (*n* in parentheses). The differences versus 1×Met or 6×Met samples were significant at * *p* < 0.05, ** *p* < 0.01, and *** *p* < 0.001 in the Student’s *t*-test.

Several behavioral tests were conducted on mice fed with control (1×Met), 6×Met, or 10×Met diet for a week to explore typical symptoms observed in schizophrenic patients, including positive/negative symptoms and cognitive/memory dysfunction. In a conventional open-field test (Figure 5A), the times (latencies) to escape the center area (Area 1) were indistinguishable (Figure 5B), but the mice on the 10×Met diet tended to spend longer time in the corner area behind the wall, Area 3 (Figure 5C), travel shorter distances (Figure 5D), and displayed a smaller number of rises (Figure 5E) compared to the control or 6×Met-fed mice. In contrast, in an elevated plus-maze test, all mice displayed equivalent dwell times in each of the open arm, closed arm, and center area (Figure 5F), although mice on the 10×Met diet were prone to spend more time in the closed arm (*p* = 0.071).

When faced with emergency/unpleasant situations, such as water soaking and tail hanging, mice on the 10×Met diet displayed more excited behavior relative to the control fed mice; they struggled for a longer time to escape from the water (Figure 5G) and tail hanging (Figure 5H). In a Y-maze spontaneous alteration test, which measured the willingness of mice to explore new environments as well as their learning/memory ability to avoid an already entered arm, 10×Met-fed mice displayed fewer arm entries, perhaps as a consequence of smaller travel distance, but the normal choice of arms that avoids the last two recently entered arms (Figure 5I).

### 2.4. Impact of High-Met Diets on Transsulfuration-Defective Cbs^+/−^ or Cth^+/−^ Mice

**Figure 6:**
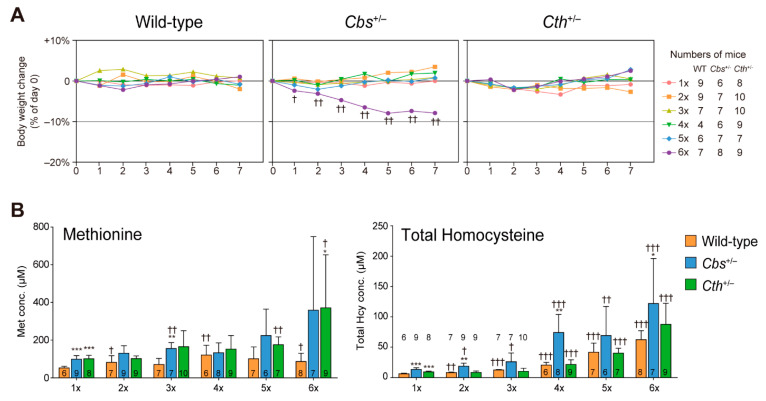
Impact of one-week high-methionine (Met) diet on body weights and serum levels of methionine (Met) and total homocysteine (Hcy) in wild-type and transsulfuration-defective mice. Adult C57BL/6J (wild-type), CBS heterozygous (*Cbs*^+/−^), and Cth heterozygous (*Cth*^+/−^) male mice were ad libitum fed a standard CE-2 rodent diet (1×Met [0.440%]) until 8–9 weeks of age and then fed 1×Met or CE-2 containing graded concentrations of Met (2×Met [0.880%]–6×Met [2.64%]) for a week. Bodyweight changes (**A**) and serum levels of Met ((**B**), left) and total Hcy ((**B**), right) are presented. The differences versus their respective 1×Met samples were significant at ^†^ *p* < 0.05, ^††^ *p* < 0.01, and ^†††^ *p* < 0.001 and versus wild-type samples at * *p* < 0.05, ** *p* < 0.01, and *** *p* < 0.001 in the Student’s *t*-test. Numbers of samples (individual mice used) are indicated.

We previously reported that the 6×Met diet for a week induced acute lethal hepatitis in mice lacking CTH (*Cth*^−/−^), demonstrating that transsulfuration played a critical role in the detoxification of excessive dietary Met [18]. The estimated incidence of type I homocystinuria (OMIM 236200) due to CBS deficiency (*CBS*^−/−^) is 1/200,000–335,000 [21]. This is consistent with an incidence rate of 1/234,300 found by newborn screening in Japan between 1977–2019, representing a total of 218 homocystinuria patients (2020 Statistical Surveys conducted by the Ministry of Health, Labour, and Welfare of Japan, Tokyo, Japan). Although cystathioninuria (OMIM 219500) by CTH deficiency (*CTH*^−/−^) is considered a benign biochemical anomaly and rarely found in general clinical settings, its reported incidence is 1/73,000–333,000 [10]. Therefore, substantial numbers of heterologous (*CBS*^+/−^ or *CTH*^+/−^) people exist and may be susceptible to diets with high-Met contents. We thus investigated the impact of one-week high-Met (2×Met–6×Met) diets on serum Met and Hcy concentrations in heterozygous *Cbs*^+/−^ or *Cth*^+/−^ mice (*Cbs*^−/−^ mice are semi-lethal [20] and *Cth*^−/−^ mice display lethal hepatitis on the 6×Met diet [18]). Except for *Cbs*^+/−^ mice fed the 6×Met diet, the high-Met diets did not influence body weight (Figure 6A). The high-Met diets did induce dose-dependent elevation of serum Met and total Hcy levels (Figure 6B,C). Serum Met and Hcy levels in *Cbs*^+/−^ (or *Cth*^+/−^) mice fed the 6×Met diet were approximately 400 µM and 100 µM, respectively, equivalent to those in wild-type mice fed the 7×Met or 8×Met diets (Figure 2B,C).

## 3. Discussion

One-carbon metabolism, in which methyl groups are transferred from Met to various substrates, plays a central role in the regulation of gene expression and the production of numerous bioactive metabolites/mediators. In addition to its usage as a protein component, Met is converted by MAT to SAM, the methyl donor of almost all (>100) methyltransferases that target numerous proteins, DNA/RNA, phospholipids, sugars, and neurotransmitters [5]. SAM is thereafter converted to SAH, which is further hydrolyzed to Hcy, a highly reactive/toxic metabolite. Hcy is then recycled back to Met via remethylation or converted to Cys via transsulfuration using CBS and CTH. Biosynthesized Cys can be protein components as well as precursors of several bioactive metabolites such as GSH, taurine, and hydrogen sulfide (H_2_S). Hence, excessive dietary intake could have substantial epigenetic and biochemical impacts.

Toxicity of high dietary Met has been most studied in rats [7]. The two-week feeding of 1.5% Met (roughly equivalent to the 3×Met or 4×Met diets in this study) caused highly elevated plasma Met levels (3240 ± 2290 µM [mean ± SD of nine rats; highly variable] compared to 168 ± 78 µM in control rats), reduced food intake (28% less than controls), and reduced body weight growth (51% less), all of which was prevented by supplementation of glycine and serine [11]. Furthermore, the two-week feeding of 3.0% Met (roughly equivalent to the 7×Met diet) induced splenic/hepatic hypertrophy in addition to retarded growth, which was also reversed by co-supplementation with glycine and serine [22,23]. We have previously investigated the impact of one-week feeding of the 3×Met [1.32%] or 6×Met [2.64%] diet on C57BL/6J wild-type mice and transsulfuration-defective *Cth^−/−^* mice with the same C57BL/6J background [18]. We observed severe phenotypes (acute lethal hepatitis) only in *Cth^−/−^* mice. In contrast, wild-type mice appeared to be normal except for slightly retarded growth and increased serum Met and Hcy levels [18]. Therefore, the current study examined diets with much higher Met contents (7×Met–13×Met) on C57BL/6J wild-type mice.

With 7×Met–13×Met diets, we observed Met dose-dependent body weight decreases (Figure 1B), hyperaccumulation of serum Met and total Hcy (Figure 2B,C), decreased serum levels of total Cys, total GSH, and taurine (Figure 2D–F) as well as increased serum levels of all eight essential amino acids, some non-essential amino acids, and two urea cycle metabolites (Figure 3). These results suggest that wild-type mice can manage some degree of Met in their diet, but there is a threshold around the 6×Met diet. In particular, extremely high-Met in diets such as 10×Met induced hyperaccumulation of Met in CSF (Figure 4) and some behavior changes (Figure 5) within a week. Furthermore, heterozygous *Cbs^+/−^* as well as *Cth^+/−^* mice displayed higher sensitivity to 1×Met–6×Met diets in serum Met and total Hcy levels (Figure 6B). It should be noted that heterozygous *CBS^+/−^* and *CTH^+/−^* people are estimated to exist at a frequency of 1/447–1/579 and 1/270–1/577, respectively (calculation from [21]). This would mean that substantial numbers of people may be somehow susceptible to high-Met foods such as ground turkey (217% recommended daily intake [RDI] in 6 oz), beef (skirt steak; 211% RDI in 6 oz), and tuna (207% RDI in 6 oz) (https://www.myfooddata.com/articles/high-methionine-foods.php, accessed on 22 December 2021). 

Hyperhomocysteinemia and deficiencies in the one-carbon metabolism components such as folic acid and vitamin B_12_ are consistent findings in schizophrenic patients [24]. There is a strong correlation between schizophrenia and gene mutation in the transmethylation enzyme such as MAT, Met synthase, methylenetetrahydrofolate reductase, catechol-*O*-methyltransferase, and DNA methyltransferase [5]. Moreover, a recent report demonstrated that significant elevation of serum Met (and some other amino acids) was found in the early stage of schizophrenia after 5.1-year treatment of antipsychotics [25]. Based on such observations, the high elevation of Met in CSF prompted us to investigate disorders in the central nervous system functions. Although our general histology on various organs did not show any alteration (data not shown), behavioral analyses in mice fed the 10×Met diet for a week revealed bipolar disorder-like negative [more time spent in the area behind the wall (Figure 5C), decreased travel distance (Figure 5D), and fewer rises (Figure 5E)] and positive symptoms [eagerness to escape from unpleasant situations like forced swimming (Figure 5G) and tail hanging (Figure 5E)] while maintaining short-term (spatial) memories (Figure 5F). Wang et al. reported that repetitive intraperitoneal injections of Met (750 mg/kg, twice a day for 7 days) into adult male Swiss Webster mice induced schizophrenia-like negative symptoms (less interaction with unfamiliar mice), positive symptoms (increased travel distance in a 40 cm × 40 cm square chamber), and cognitive symptoms (impairments in recognition of novel objects and memory of critical situations) [6]. Soares et al. demonstrated that subcutaneous injections of Met (0.2 to 0.4 g/kg depending on the developmental stage, twice a day from postnatal day 6 to 28) into male/female Wister rats reduced grooming and rearing, but not locomotion in an open-field test, and impaired short-term memories (in both the novel object recognition and Y-maze tests) [9]. Large discrepancies could be related to the differences in animal species/strains, Met administration methods, doses and terms, experimental procedures, and base diet compositions, although the underlying molecular mechanisms remain unknown.

In summary, this study provided an animal disease model for bipolar disorder, which is easy (ad libitum feeding), quick (one-week), and less expensive (only Met and the standard diet needed) to produce and applicable to various tests using any genetically modified mice based on the (standard) C57BL/6J genetic background. Furthermore, this study raises the additional concern that substantial numbers of people (such as *CBS^+/−^* and *CTH^+/−^*) could be somehow vulnerable to high-Met foods.

## 4. Materials and Methods

### 4.1. Mice and Diets

C57BL/6J (wild-type) male mice were purchased from Japan SLC (Shizuoka, Japan). CBS heterozygous (*Cbs*^+/−^; B6.129P2-*Cbs*^tm1Unc/J^) mice [26] were obtained from the Jackson Laboratory (Bar Harbor, ME, USA) and backcrossed for 14 generations to the C57BL/6J mice. CTH heterozygous (*Cth*^+/−^) mice were generated by our group [27] and backcrossed for 11 generations to C57BL/6J. Thus, the mice were comparably analyzed on almost the same C57BL/6J background. The mice were housed in an air-conditioned room kept on a 12-h dark (8 p.m.–8 a.m.)/light cycle and allowed free access to water and diets. The standard dry rodent diet CE-2 (CLEA Japan, Tokyo, Japan) containing 0.44% Met was used as the control diet. For the preparation of high-Met diets (1.2×Met–13×Met), CE-2 powders were mixed with graded doses of l-Met (Fujifilm–Wako, Osaka, Japan), kneaded to small pellets, air-dried, and then sterilized with γ-ray irradiation (30 kGy).

### 4.2. Measurement of Serum and CSF Amino Acids

Male mice (8–11 weeks of age) were anesthetized with isoflurane inhalation. Blood was collected by cardiocentesis, and serum was prepared after coagulation and centrifugation. CSF samples were collected by ventricular puncture with glass capillaries. Serum and CSF amino acid levels were measured using high-performance liquid chromatography (HPLC), amino acid labeling with 4-fluoro-7-nitrobenzofurazn (NBD-F; Dojindo, Kumamoto, Japan), and thiol labeling with 4-fluoro-7-sulfobenzofurazn (SBD-F; Dojindo) as previously described [28,29]. NBD-F labeling of tryptophan (Trp) resulted in a fluorescence wave change, and Trp was detected by its fluorescence (excitation: 295 nm; emission: 340 nm). Asparagine was not measurable because its peaks were too close to those of NBD-F derivatives. Because the minimum volume required for all our assays (NBD-F labeling, SBD-F labeling, and Trp detection) is 8 µL (the routine serum volume used was 25 µL), equal CSF volumes (3–5 µL) of two–three mice were pooled. For the detection of thiol-containing amino acids, serum and CSF samples were reduced to cleave disulfide bonds before SBD-F labeling, and the total levels of Hcy, Cys, and GSH (tHcy, tCys, and tGSH, respectively) were measured [28,29].

### 4.3. Serum Biochemistry

Serum levels of AST, ALT, LDH, GLU, BUN, and TG were measured using a Dri-Chem 7000i clinical analyzer (Fuji Film, Osaka, Japan). Serum levels of lipid peroxidation (thiobarbituric acid reactive substances [TBARS] such as MDA) were examined using a TBARS assay kit (Cayman Chemical, Ann Arbor, MI, USA).

### 4.4. Behavior Analyses

Male mice (8–11 weeks of age) were fed the control (1×Met) CE-2, 6×Met, or 10×Met diet for a week before behavior analyses. All behavioral activities were recorded using NVCap software (freeware from Net vision, Tokyo, Japan) or an AGDRec desktop recorder (freeware from T. Ishii’s Software Library, Osaka, Japan) and analyzed by Fiji (National Institutes of Health, Bethesda, MD, USA).

#### 4.4.1. Open-Field Test

The open-field test was performed to assess locomotor and exploratory activities as previously described [20]. Each mouse was placed in the center of an “open-field” (a round-shaped arena, 48 cm in diameter surrounded by 20 cm high walls made of light blue acrylic board) and allowed to roam the field for 5 min under video recording. The time taken to exit Area 1, retention times in the center (Area 1), side (Area 2), and corner (Area 3) areas (1/3 each of the total area), and distances traveled were measured and the number of rises was counted in the video.

#### 4.4.2. Elevated Plus-Maze Test

The elevated plus-maze test was performed as previously described [20]. The apparatus consisted of four arms (25 × 6 cm), two enclosed by 16 cm high walls and two exposed, elevated 50 cm off the ground. Each mouse was placed in the center zone facing the same open arm. The amount of time spent in the open areas (two arms and the center region, excluding the two high-wall arms) during the 5-min test was determined as a measure of anxiety.

#### 4.4.3. Forced Swim Test

The forced swim test was conducted as previously described [6]. Each mouse was placed in a transparent acrylic cylinder containing water (25 cm high, 10 cm diameter, 15 cm water depth) at 25 °C and forced to swim for 7 min under video recording. The immobility time was recorded as a measure of despair/depression [30,31].

#### 4.4.4. Tail Suspension Test

The tail suspension test was performed as previously described [32]. Each mouse was suspended by its tail with tape for 6 min and the immobility time was recorded as another measure of despair/depression.

#### 4.4.5. Y-Maze Spontaneous Alteration Test

The Y-maze test was performed to measure exploratory activity and short-term (spatial) memory [33,34]. A Y-maze apparatus consisting of three identical arms (30 × 6 × 15 cm, 120° apart) and the center region, all made of gray acrylic plates, with clues located in the periphery of the room to allow visual orientation was used. Each mouse was placed at the end of one arm and allowed to move freely through the maze for 5 min. Alternation behavior was defined as consecutive entries into each of the three arms without repetition (i.e., ABC, BCA but not ABA, BCB). Spontaneous alternations (in percentages) were defined as the number of correct alternations divided by the number of total alternations (total arm entries minus 2) × 100. Total arm entries were scored as an index of exploratory activity and the number of correct choices as an index of short-term memory.

## Data Availability

Not applicable.

## Supplementary Materials

The following is available online at https://www.mdpi.com/article/10.3390/ijms23020928/s1.
