# Supplementary material for: A High-Methionine Diet for One-Week Induces a High Accumulation of Methionine in the Cerebrospinal Fluid and Confers Bipolar Disorder-like Behavior in Mice
Source: Int J Mol Sci. 2022 Jan 15;23(2):928. doi: 10.3390/ijms23020928 (PMC8778486; doi:10.3390/ijms23020928)
Supplement: Supplementary file 1 [file ijms-23-00928-s001.zip › ijms-1546144-supplementary.pdf]

Supplementary Table S1. Serum biochemical parameters in mice fed with 1×Met–13×Met diets for a week.

Diet	AST (U/L)	ALT (U/L)	LDH (U/L)	GLU (mg/dL)	BUN (mg/dL)	TG (mg/dL)	MDA (μM)
1×	71 ± 12 (4)	22 ± 8 (6)	248 ± 127 (4)	285 ± 70 (4)	27 ± 5 (6)	103 ± 38 (3)	2.7 ± 0.6 (4)
1.2×	91 ± 52 (4)	20 ± 6 (4)	not tested	389 ± 84 (4)	34 ± 13 (4)	not tested	3.5 ± 1.2 (4)
1.4×	99 ± 9 (4) *	20 ± 2 (4)	not tested	430 ± 34 (4) *	24 ± 1 (4)	not tested	2.5 ± 0.2 (4)
1.6×	109 ± 46 (4)	22 ± 2 (4)	not tested	312 ± 64 (4)	28 ± 2 (4)	not tested	2.7 ± 0.2 (4)
1.8×	99 ± 46 (4)	20 ± 5 (4)	not tested	358 ± 37 (4)	30 ± 2 (4)	not tested	2.7 ± 0.4 (4)
2×	119 ± 30 (4) *	42 ± 17 (4)	not tested	426 ± 65 (4) *	30 ± 4 (4)	not tested	2.7 ± 0.8 (4)
3×	128 ± 47 (4)	27 ± 7 (4)	not tested	366 ± 43 (4)	30 ± 4 (4)	not tested	2.3 ± 0.2 (4)
4×	121 ± 48 (4)	33 ± 12 (4)	not tested	367 ± 59 (4)	29 ± 5 (4)	not tested	2.9 ± 1.2 (4)
5×	104 ± 35 (3)	42 ± 22 (4)	not tested	399 ± 70 (3)	28 ± 3 (3)	not tested	2.2 ± 0.3 (4)
6×	116 ± 50 (4)	36 ± 19 (4)	298 ± 8 (4)	314 ± 47 (4)	25 ± 3 (4)	not tested	2.9 ± 1.1 (4)
7×	71 ± 32 (3)	30 ± 6 (3)	321 ± 166 (4)	309 ± 90 (4)	24 ± 4 (4)	not tested	2.2 ± 0.3 (4)
8×	87 ± 19 (4)	36 ± 13 (4)	203 ± 27 (4)	271 ± 53 (4)	25 ± 3 (4)	not tested	2.2 ± 0.3 (4)
9×	101 ± 38 (3)	148 ± 150 (4)	362 ± 167 (4)	255 ± 41 (4)	24 ± 4 (4)	not tested	1.8 ± 0.5 (4)
10×	109 ± 37 (3)	255 ± 388 (4)	406 ± 259 (4)	214 ± 27 (4)	31 ± 5 (4)	118 ± 80 (4)	1.8 ± 0.2 (4) *
11×	118 ± 35 (4)	82 ± 35 (4) *	324 ± 45 (4)	227 ± 48 (4)	37 ± 5 (4) *	116 ± 78 (4)	1.8 ± 0.0 (4) *
12×	250 ± 240 (3)	172 ± 114 (4)	445 ± 114 (3)	184 ± 32 (4)	36 ± 2 (3) **	140 ± 104 (4)	2.0 ± 0.7 (3)
13×	99 ± 20 (3)	139 ± 189 (4)	316 ± 62 (3)	154 ± 35 (4) *	36 ± 7 (4)	37 ± 12 (4)	1.6 ± 0.1 (4) *

Serum levels of aspartate aminotransferase (AST), alanine aminotransferase (ALT), lactate dehydrogenase (LDT), glucose (GLU), blood urea nitrogen (BUN), triglyceride (TG), and malondialdehyde (MDA) were measured. Data are mean ± SD (sample numbers). Differences *versus* the control (1×Met) samples are significant at $*P < 0.05$ and $**P < 0.01$ in the Student's *t*-test. Some samples were not tested for LDH and TG because no apparent alterations were observed in the diets with much higher Met doses.
